# The Impact of Inclusive Talent Development Model on Turnover Intention of New Generation Employees: The Mediation of Work Passion

**DOI:** 10.3390/ijerph17176054

**Published:** 2020-08-20

**Authors:** Yang-Chun Fang, Jia-Yan Chen, Xu-Dong Zhang, Xin-Xing Dai, Fu-Sheng Tsai

**Affiliations:** 1 Global Institute for Zhejiang Merchants Development, Zhejiang University of Technology, Hangzhou 310023, China; 2 Department of Psychology and Behavioral Sciences, Zhejiang University, Hangzhou 310023, China; 3 The School of Management, Zhejiang University of Technology, Hangzhou 310023, China; cjy17816873275@163.com (J.-Y.C.); xudong_zhang_work@163.com (X.-D.Z.); Elaine19880914@126.com (X.-X.D.); 4 North China University of Water Resources and Electric Power, Zhengzhou 450046, China; 5 Department of Business Administration, Cheng Shiu University, Kaohsiung 833, Taiwan; 6 Center for Environmental Toxin and Emerging-Contaminant Research, Cheng Shiu University, Kaohsiung 83347, Taiwan; 7 Super Micro Mass Research and Technology Center, Cheng Shiu University, Kaohsiung 83347, Taiwan

**Keywords:** inclusive talent development model, new generation employees, questionnaire investigation, turnover intention, work passion

## Abstract

The high turnover rate of new generation employees is becoming an increasingly important issue for business and academia. Based on self-determination theory and conservation of resource theory, our study explores the impact of the inclusive talent development model on the turnover intention of new generation employees and the mediating role of work passion. Based on the questionnaire of 290 new generation employees’ data from China, after common method biases and reliability and validity tests, we used SPSS, Mplus and bootstrapping for empirical analysis. The result showed that the inclusive talent development model includes the diversified talent team construction, rational tolerance of employee’s opinions and failures, emphasis on employee’s training, emphasis on fairness and win–win and emphasis on employee’s advantages. The work passion has been divided into harmonious passion and obsessive passion. Among them, rational tolerance of employee’s opinions and failures, emphasis on employee’s training and emphasis on fairness and win–win have a significant positive impact on harmonious passion. Emphasis on fairness and win–win and emphasis on employee’s advantages have negative correlation with obsessive passion. The employee’s harmonious passion is significantly negatively correlated with the turnover intention and the obsessive passion is significantly positively correlated with the turnover intention. In addition, the harmonious passion plays a mediating role between rational tolerance of employee’s opinions and failures, emphasis on employee’s training, emphasis on fairness and win–win and employee’s turnover intention, while the obsessive passion plays a mediating role between emphasis on fairness and win–win and emphasis on employee’s advantages and employee’s turnover intention.

## 1. Introduction

The new generation employees have gradually become the main part of the labor force. They have gradually become the main part of employees of enterprises because of their pursuit for freedom and distinctive personality. The new generation refers to people who were born between 1980 and 1999. It is also referred to as Generation Y or Millennials [1]. While new generation employees refers to the young practitioners who were born after 1980 and have the ability to work, the new generation employees are young practitioners who were born after 1980 and have already had the ability to work. Compared with the old generation employees, the new generation employees generally have lower organizational loyalty and higher frequency of job hopping [2]. Park and Shaw have pointed out that employees’ frequent turnover behaviors will lead to internal knowledge leakage and loss of the important customers [3]. In other words, frequent turnover behaviors of new generation employees will bring some problems to business management. What we should address is the problem of how organizations attract and retain new generation employees and weaken their willingness to quitting [4]. The organizational workplace plays a very important role in influencing new generation employees’ work attitude and behaviors. Enterprise managers can formulate strategies to address the concerns and needs of new generation employees [5]. For this reason, we cannot help thinking about what management practices managers should adopt to manage effectively the turnover of the new generation employees. With the increase in talent demand and the change of organizational structure, more and more organizations need to integrate the inclusive concept into modern management. In management practice, “Inclusion” is an important part of Chinese culture. It is not only a high-quality culture, but also an important management concept. Inclusive organizational management practices are accepted more easily by employees and organizations.

Inclusion is the historical enlightenment of the Chinese civilization [6]. It is also the symbol of a modern civilized society [7], and an important concept of the UN Millennium Development Goals. Inclusive development is the trend of the world [8]. In recent years, scholars pay more and more attention to the significance of the concept of inclusiveness to modern organization management, emphasizing that organizations need to carry out inclusive innovation and achieve inclusive growth [9]. The idea of inclusiveness builds diverse workforce’s support to improve the working environment and standard [10]. The diverse management form has developed into inclusive form [11]. More and more scholars had proposed that organizational context factors such as inclusive organizational atmosphere and inclusive leadership can affect employee’s perception of tolerance, and further affect organizational citizenship behavior, employee turnover, etc. [10,12]. Hwang and Hopkins had pointed out that when the organization adopts a more inclusive management model, employees can have organizational commitment and improve job satisfaction, and decrease their turnover intention [13]. The inclusive talent development model has become an emerging theme in the field of human resource management in recent years [14]. In order to enable different individuals to participate more fully and provide opportunities for all members of the organization to realize their potential, researchers and practitioners increasingly use inclusion as a way to achieve these goals [15]. Inclusion is an important environmental factor for retaining talents and making good use of them. Therefore, talent development must be “inclusive” [16]. The inclusive talent development model incorporates organically the concept of tolerance into the enterprise’s human resource management practice. It is an organic fusion of traditional “inclusive culture” and modern “inclusive concept” into a series of talent development work such as attracting, employing, educating and motivating the talent [17]. The difference from the traditional human resource management practice is that the inclusive talent development model is not limited to the traditional module of human resource management. It focuses more on the effective management and the employee’s cultivation by building an inclusive organizational atmosphere to achieve the sustainable development of employees and organizations. More importantly, the inclusive talent development model is a series of talent development work taken by the organization in order to build an inclusive working environment and provide high-quality working resources for its employees. It emphasizes five aspects, including the heterogeneity of inclusive talents, introducing talents in a diversified way; employing talents, emphasizing the advantages of employees; treating employees fairly, achieving a win–win situation between employees and the organization; emphasizing educating talents and their growing process; rational and inclusivity of employee’s views and failures, and encouraging talent innovation. The inclusive talent development model had been proved to have a significant impact on organizational innovation performance [18], innovation passion [19], employee craftsman spirit [20] and employee work engagement [21], etc. Besides, it has the remodeling effect on the work [22]. Empirical research of the inclusive talent development model is still in its infancy currently. In the past studies, scholars have studied the antecedent variables of employees’ turnover intentions mainly in regard to job satisfaction, job embedding, organizational commitment and organizational support. Research about the impact of inclusive management practices, especially the inclusive talent development model on employee turnover, is relatively lacking [8].

In view of the complexity of the mechanism of organizational inclusive management and employee behavior, it is necessary to study the impact of the inclusive talent development model on the turnover intention of employees in the Chinese context and uncover the “black box” of the inclusive talent development model’s effects. It is worth stressing that the data of the workplace survey on “work passion” from 51job (www.51job.com) in 2017 showed that up to 97.5% of people in the workplace are more or less facing the loss of work passion, and only 2.5% of the respondents said that they always maintain work passion. Vallerand and Houlfort had pointed out that work passion is the strong tendency or willingness of members of an organization to work [23]. That means individuals like, or even love, their work—therefore, investing time and energy—and regard the work as a self-identified core identity characteristic. In addition, dualistic work passion is a motivation structure that can be divided into harmonious passion and obsessive passion. Among them, harmonious passion is related to individual internalization. Individuals can voluntarily consider work as an important part of their identity, while obsessive passion is related to the internalization of individual’s controlled forms of internal or interpersonal pressure. Recent research has pointed out that work passion is the unique explanatory power of variables such as occupational happiness and burnout. This can help us to understand the phenomenon that other variables cannot be thoroughly interpreted in previous studies [24]. Some scholars had suggested to stimulate individual work passion from the organizational management perspective [25]. Based on this, we will pay attention to the question of whether the inclusive talent development model will have an impact on employee’s work passion and then affect their turnover intentions or not. In other words, we will analyze the mediation of work passion between the inclusive talent development model and the turnover intention of new generation employees, aiming to further improve and develop the theoretical framework of inclusive management research, as well as the human resource development model and employee behavior research. The hypotheses put forward in this study take work passion as the mediating variable between the inclusive talent development model and employee turnover intention, incorporating the “work passion” factor into the study, which enriches the existing variable research on the inclusive talent development model, and provides a theoretical basis for the inclusive human resource management practice of enterprises.

## 2. Literature and Hypothesis Development

### 2.1. Inclusive Talent Development Model and New Generation Employees’ Work Passion

Traditional enterprise talent development refers to the exploration and cultivation of an employee’s human capital as a resource in order to improve their professional skills and quality. The talent development model refers to the methodology of solving such problems [20]. 

The relationship between the inclusive talent development model and the work passion of new employees can be explained by the self-determination theory. This theory states that the three basic psychological needs of competence, relatedness and autonomy are innate. All individuals work hard to meet these needs and they tend to the environment which can meet these needs [26]. Competence means that individuals can successfully complete challenging tasks and achieve their desired results [27]. Autonomy means that individuals can choose and make decisions on their own and perceive that they are creators of actions. Relatedness means individuals need to establish the feelings of mutual respect and dependence with others [28]. The inclusive talent development model includes the five dimensions: diversified talent team construction, rational tolerance of employee’s opinions and failures, emphasis on employee’s training, emphasis on fairness and win–win and emphasis on employee’s advantages. This model can meet an individual’s three basic psychological needs. Specifically, diversified talent team construction means that organizations accept diverse talents and are willing to increase the work participation and authority of diverse employees. Some studies have pointed out that the diversity of employees, such as gender, age, and ethnicity, has a strong impact on negative emotions such as organizational exclusion in the workplace [13]. The adoption of a diverse talent team is conducive to improving employee perception and organization In the same way, rationally tolerating employees’ opinions and failures does not only ensure that employees do not have to be punished when they suffer a defeat, but also relieves the negative and pessimistic emotions caused by job failures, consequently enhancing the likelihood of employees supporting and respecting the organization. Emphasis on an employee’s training can help employees improve their personal professional skills so as to better complete challenging tasks in the organization. Emphasis on fairness and win–win results can provide employees a fairer experience and ensure that employees can get expected results when they complete organizational tasks. Emphasis on the use of employee’s advantages means the organization arranges work and function authorization reasonably according to the advantages of employees so as to improve the identity perception and autonomous experience of employee’s inside identity.

According to the self-determination theory, the inclusive talent development model gives employees a certain job autonomy which can affect their work passion. Some scholars have pointed out that external control factors such as deadlines and mandatory tasks and so on will have a certain impact on an employee’s perceptions of autonomy and then influence their passion for the job [29]. In addition, job autonomy can also be reflected in the decision-making process of employees during work which reflects the extent to which employees can complete tasks through decision opportunities and control in their careers. Deci and Ryan had pointed out that autonomous environment has promoted the employee’s process of internalization of activities [28]. This helps employees see their behaviors more freely and express their opinions at work. In other words, work autonomy will facilitate the process of individual internalization. Due to the strong autonomy of the new generation employees, individuals naturally tend to take the initiative actions and look for fun and challenging activities. Therefore, job autonomy will promote the harmonious passion of new employees more easily. In contrast, a low work autonomy environment promotes an individual’s opinion that they are controlled, which is further internalized by external factors [26]. Individuals lack the opportunity to make decisions on important tasks and they have to abide by and deal with external tasks which do not meet their goals and values. This will destroy the harmony and produce conflicts during employee’s self-internalization process which will cause obsessive work passion. Based on the above analysis, this study proposed the following hypotheses:

**Hypothesis 1 (H1):**  The inclusive talent development model (**H1a:** rational tolerance of employee’s opinions and failures. **H1b:** diversified talent team construction. **H1c**: emphasis on employee’s training. **H1d:** emphasis on fairness and win–win. **H1e:** emphasis on employee’s advantages.) has a positive impact on the harmonious passion of new generation employees.

**Hypothesis 2 (H2):**  The inclusive talent development model (**H2a:** rational tolerance of employee’s opinions and failures. **H2b:** diversified talent team construction. **H2c:** emphasis on employee’s training. **H2d:** emphasis on fairness and win–win. **H2e:** emphasis on employee’s advantages.) has a negative impact on the obsessive passion of new generation employees.

### 2.2. Work Passion and New Generation Employees’ Turnover Intentions

Previous research has shown that work passion can have a significant impact on employees’ attitudes and behaviors [1]. This study aims to explore whether both harmonious passion and obsessive passion can predict the turnover intention of new generation employees. The turnover model indicates that employees have different reasons to leave their jobs, but they are all affected by external factors such as the reward system in the organization, the job’s needs and relationship with colleagues more or less [30]. According to the self-determination theory, the degree to which an individual is affected by external factors depends on the degree to which the individual internalizes the activity. Although both harmonious passion and obsessive passion can be affected [31], the degrees to which they are affected by external factors are different. Harmonious passion is derived from individual cognition and positive emotions. This passion is affected less by external factors [32]. So, employees with harmonious passion can freely control their decisions and behaviors at work. Some studies have shown that a sense of job control can weaken an employee’s turnover intention [33]. So, we predict that harmonious passion is negatively related to employee turnover intention. In contrast, individuals with obsessive passion tend to think that they are working in an environment controlled by external control [23]. Due to the existence of external control factors, these employees may feel pressure to work a specific way so as to reduce external control to leave. So, it can be predicted that obsessive passion is positively related to employee’s turnover intention. Previous studies have also demonstrated the relationship between work passion and related variables of turnover intention. The research by Vallerand found that harmonious passion actively predicts job satisfaction of employees and suppresses employee job burnout. However, obsessive passion will lead employees to produce more job conflicts and job burnout [34]. Previous studies have shown that job satisfaction and job burnout can significantly affect employee’s turnover intention. Based on the above analysis, this study proposes the following hypotheses:

**Hypothesis 3 (H3):**  The harmonious passion of new generation employees has a significant negative impact on the employee’s turnover intention.

**Hypothesis 4 (H4):**  The obsessive passion of new generation employees has a significant positive impact on the employee’s turnover intention.

### 2.3. The Mediation of Work Passion

Most studies have found that work passion has a mediation impact on related variables in the studies. For example, Xu and his team had discovered the mediation of dualistic work passion in abusive supervision and subordinate voice behavior [35]. Similarly, some scholars had explored dualistic work passion mediates between enterprises’ ethical leadership and employee’s voice [36]. Based on previous research and the above discussions on the inclusive talent development model and work passion, or work passion and turnover intention, we speculate that the inclusive talent development model and the new generation employees’ turnover intentions may be mediated by dualistic work passion. Specifically, diversified talent team construction, rational tolerance of employee’s opinions and failures and emphasis on employee’s advantages can promote employee’s perception of organizational tolerance and work autonomy. According to the self-determination theory, in an autonomous work environment, employees are likely to internalize external work as a part of their identity. This will lead employees to have a harmonious passion and to do the work in a full state [26]. On the contrary, a self-support environment can reduce employee’s perception of external control and suppress the generation of obsessive passion. Previous studies have shown that harmonious passion can improve employee’s job satisfaction and then relieve job burnout [37]. Studies by related scholars have shown that a burnout model can positively predict the turnover intention of employees [38]. Emphasis on employee’s training and emphasis on fairness and win–win can lead employees to experience more work resources. Resource conservation theory states that individuals have the instinct to acquire, maintain, protect and cultivate valuable resources [39]. So, when employees experience valuable work resources through organizational inclusive management, they will work harder to protect and cultivate these resources. It should be pointed out that the inclusive talent development model can provide employees with more work resources [20]. This can stimulate an employee’s harmonious passion and suppress an employee’s obsessive passion. From the perspective of resource conservation theory, when new generation employees have harmonious passion, they are more inclined to devote rich resources into a job to protect, cultivate, and acquire more resources rather than leaving the job to lose work resources [39]. Conversely, although individuals with obsessive passion will be engaged in a certain job, they will feel the control and the loss of their own resources [40]. So, they will generate turnover intention to avoid possible resource loss in the future. Based on the above analysis, it can be considered that the impact of the inclusive talent development model on an employee’s turnover intention can be achieved through the special mechanism of dualistic work passion. The following hypotheses are made:

**Hypothesis 5 (H5):**  The harmonious passion of new generation employees plays a mediating role between the inclusive talent development model (H5a: rational tolerance of employee’s opinions and failures. H5b: diversified talent team construction. H5c: emphasis on employee’s training. H5d: emphasis on fairness and win–win. H5e: emphasis on employee’s advantages.) and turnover intention of new generation employees.

**Hypothesis 6 (H6):**  The obsessive passion of new generation employees plays a mediating role between the inclusive talent development model (H6a: rational tolerance of employee’s opinions and failures. H6b: diversified talent team construction. H6c: emphasis on employee’s training. H6d: emphasis on fairness and win–win. H6e: emphasis on employee’s advantages.) and turnover intention of new generation employees.

**Figure 1:**
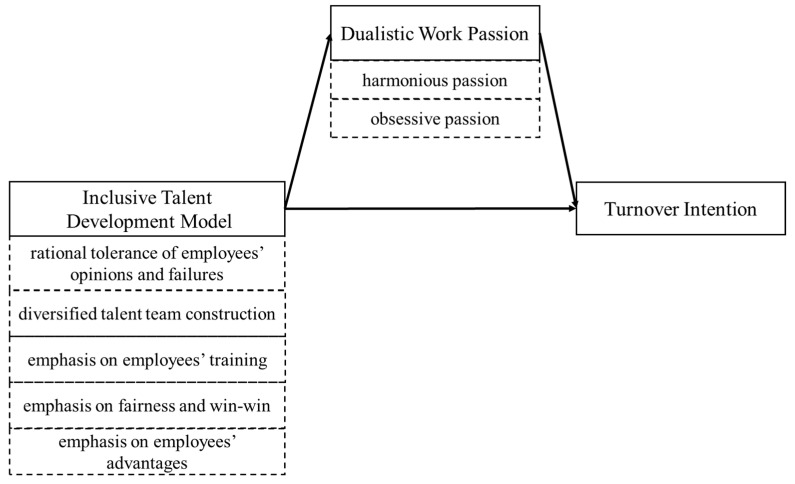
Research hypothesis model.

We summarized the hypotheses into a research model in the Figure 1. 

## 3. Research Design

### 3.1. Sample Participants

The target of this questionnaire was the new generation employees. The respondents were from enterprises in the Yangtze River Delta region such as Zhejiang, Jiangsu, and Anhui. The Yangtze River Delta is one of the most developed regions in the country’s economy. In 2019, the GDP of the Yangtze River Delta totaled CNY 23.7 trillion, accounting for 23.9% of the national total. In addition, it is also a region where new generation employees gather. According to the “Research Report on digital economy and talent development in the Yangtze River Delta” jointly released by LinkedIn, Center for Internet Development and Governance of Tsinghua SEM (School of Economics and Management) and Shanghai Institute of Science and Technology Policy, the Yangtze River Delta region is more attractive for talents than other regions in the world, including Hong Kong, Macao, Taiwan and other regions in China, and is the preferred working area for young people. Therefore, taking the new generation in the Yangtze River Delta as the research object has certain representativeness.

The ratio of male and female is balanced with men accounting for 53.8% and women accounting for 46.2%. In terms of age, the proportion of employees in the age group of 18 to 24 and 24 to 29 has reached 65.2%. This age group is also the main force of the new generation of employees, and because the data were from the Yangtze River Delta region, which is the first choice of the younger generation, our research objective has representative significance. In terms of education level, the undergraduate degree accounted for the highest proportion, accounting for 48.6% of the total number of people. Bachelor’s degree or above accounted for more than 77%. In terms of working years, the proportion of individuals with a working age of 1 year has reached 12.8%. Additionally, the proportion of employees with one to three years’ working experience has reached 29.0%. Those working for three to five years represent 32.8% of all these employees. In terms of job level, the number of general staff and grassroots administrators account for 50.3% and 36.2% of the total number. The above data can reflect the characteristics of the survey group, which is in line with the overall distribution of the characteristics of the new generation of employees. From the data, these young new generation of employees often lived in grassroots positions, with higher education and short-term employment, which is the most mobile group in the enterprise. The research on this group in the Yangtze River Delta is more targeted and representative. The detailed descriptive data of the study sample are provided in Table 1.

### 3.2. Method and Variables

In this study, the inclusive talent development model is a 15-item questionnaire that was designed from previous research and interviews with typical companies. The scale is divided into five dimensions and the Cronbach’s α value of each subscale is greater than 0.7. So, the inclusive talent development model scale has a good reliability. The work passion scale is a 14-item scale designed by Vallerand [27]. The work passion scale is divided into two dimensions including harmonious passion and obsessive passion. The Cronbach’s α value of each subscale is 0.886 and 0.883. So, the work passion scale has a good reliability. The turnover intention scale is a 4-item scale designed by Farh [41]. The scale is a single-dimension scale with a Cronbach’s α value of 0.828. Therefore, the turnover intention scale has a good reliability. Table 1 shows the related indexes of scale reliability and convergence validity test. The control variables in this study were gender, age, educational background, working experience and job rank.

### 3.3. Procedure

#### 3.3.1. Data Collection

The questionnaires were mainly distributed to enterprises in Zhejiang, Jiangsu, Anhui and other places by combining online and offline. Among the two ways, online distribution was mainly achieved through online questionnaire platforms. In the process of offline distribution, the questionnaires were mainly carried out in the following two ways: one was that the interview group was distributed through the training courses of the school’s MBA (master of business administration) education center for on-site distribution and recycling; the other was that during the field visit to enterprises, questionnaires were conducted within the enterprise issues. The data collection activities were mainly concentrated from May 2019 to August 2019, and a total of 320 questionnaires were collected. In the process of screening the questionnaires, the questionnaires that were filled randomly, missed, and those with age characteristics that did not meet the new generation employees were deleted. Finally, 290 valid questionnaires were retained with an effective recovery rate of 90.6%.

#### 3.3.2. Common Method Bias Test

In this study, the survey questionnaires were filled by the same one person during the process of issuing questionnaires, so the problem of common method bias could not be eliminated. Therefore, this study uses Harman’s single factor test to detect common method bias. In previous studies, all the items in the questionnaire were put into exploratory factor analysis and the problem of common method bias was judged by judging the factors that were precipitated or by judging that the explanatory power of a certain factor was particularly strong. In this study, the analysis results show that the amount of interpretation of the first factor that is precipitated is 29.71%. It is far below the critical value of 40% and there is no single factor. So, the problem of common method bias in this study is not serious.

## 4. Data Analysis Results

### 4.1. Descriptive Statistics and Related Analysis

The results of descriptive statistics are shown in Table 2. The average score of five dimensions of the inclusive talent development model is shown: Rational tolerance of employee’s opinions and failures scores 3.37; Diversified talent team construction scores 3.24; Emphasis on employee’s training scores 3.41; Emphasis on fairness and win–win scores 3.1; Emphasis on employee’s advantages scores 2.95.

In the inclusive talent development model perceived by employees, rational tolerance of employee’s opinions and failures, emphasis on employee’s training and diversified talent team construction achieve higher level. This means that the leaders of surveyed enterprises can conduct diversified talent team construction to ensure a diversity of employees. During the career of new employees, relevant leaders can better tolerate the opinions and failures of their subordinates and attach importance to the training of new employees to help them grow into talents and ensure their rapid growth in the enterprise. The emphasis on fairness and win–win and the emphasis on employee’s advantages in the situation perceived by employees doesn’t reach a high level. This indicates that relevant companies still have room for improvement in creating a fair organizational atmosphere and the use of employee’s advantages.

Before conducting regression analysis and testing of mediating effects, the correlation between variables should be examined. It can be seen from Table 3 that all dimensions of the inclusive talent development model are significantly negatively correlated with turnover intention, while each dimension of the independent variables is significantly correlated with the dependent variable. That lays the foundation for subsequent regression analysis and testing of the mediating effects. In addition, the dimensions of the inclusive talent development model are significantly positively correlated to the harmonious passion of new generation employees and significantly negatively correlated to obsessive passion of employees. This means that when the inclusive talent development model is adopted more effectively by the organization, the harmonious passion of new generation employees is higher and obsessive passion is lower. Based on the above analysis, it can be assumed that H1 and H2 may hold. Harmonious passion is significantly negatively correlated with employee turnover intention. This means that the higher the level an employee’s harmonious passion is, the more likely they are to suppress the generation of turnover intention. This result initially supports H3 of this paper. On the contrary, obsessive passion has a significant positive impact on the level of an employee’s turnover intention. That means the employees with higher level of obsessive passion are more likely to have the intention to leave the organization. This initially supports the hypothesis H4.

### 4.2. Regression Analysis

In order to study the impact of the dimensions of the inclusive talent development model on employee’s dualistic work passion and turnover intention and the impact of the dualistic work passion on employee’s turnover intention, this paper used the regression analysis method. This method takes harmonious passion and obsessive passion and employee’s turnover intention as the dependent variable and analyzes the role of inclusive talent development model variables, work passion and employee’s turnover intention. The results are shown in Table 4. Equations 1-1 and 1-2 in Table 4 use harmonious passion as the outcome variable for regression. The results show that the five factors of the inclusive talent development model entering the regression equation can explain 32.8% of the total variation. F1 (rational tolerance of employee’s opinions and failures), F3 (emphasis on employee’s training) and F4 (emphasis on fairness and win–win) have a significant positive impact on employee’s harmonious passion, while F2 (diversified talent team construction) and F5 (emphasis on employee’s advantages) have no significant impact on employee’s harmonious passion. Therefore, H1a, H1c and H1d are established but H1b and H1e do not pass the test. Equation 2-1 and Equation 2-2 perform stepwise regression with obsessive passion as the dependent variable. The results show that the five factors of the inclusive talent development model entering the regression equation can explain 27.4% of the total variation. Among them, F1 (rational tolerance of employee’s opinions and failures), F2 (diversified talent team construction) and F3 (emphasis on employee’s training) have no significant impact on employee’s obsessive passion, while F4 (emphasis on fairness and win–win) and F5 (emphasis on employee’s advantages) have a significant negative impact on employee’s obsessive passion. Therefore, H2d and H2e are established but H2a, H2b and H2c not. In the regression analysis with turnover intention as the dependent variable, this study examined the impact of each dimension of the inclusive talent development model on employee’s turnover intention and the impact of dualistic work passion on employee’s turnover intention. Equation 3-1 and Equation 3-2 use dualistic work passion as the independent variable to analyze the turnover intention. The results show that harmonious passion has a significant inhibitory effect on employee’s turnover intention, while obsessive passion has a significant positive impact on employee’s turnover intention. The dualistic work passion factors entering the regression equation can increase the explained variation by 25.1%. Therefore, H3 and H4 pass the data test. Equation 4-1 and Equation 4-2 analyze the employee’s turnover intention with the five factors of the inclusive talent development model as independent variables. The results show that F1 (rational tolerance of employee’s opinions and failures), F3 (emphasis on employee’s training), F4 (emphasis on fairness and win–win) and F5 (emphasis on employee’s advantages) all have a significant negative impact on employee’s turnover intention. However, F2 (diversified talent team construction) has no significant impacts on turnover intention of new generation employees.

### 4.3. Mediation Test

Previous studies have shown that Mplus has a better inspection effect on dealing with complex process models. So, this research selected Mplus7.4 as the analysis software to test the mediating role of harmonious passion and obsessive passion between each dimension of the inclusive talent development model and employee’s turnover intention. This study estimates parameters and tests the mediation through Mplus program and setting Bootstrap (N = 1000). Table 5 shows that all the fitting indices of the mediating model meet the fitting criteria. It indicates that the model data fit well, and the model results are acceptable.

The results of the mediation effect test are shown in the Table 6. In the inclusive talent development model of an enterprise, rational tolerance of employee’s opinions and failures can have an impact on employee’s turnover intention through harmonious passion with an intermediary effect of −0.038. It does not contain 0 within a 95% confidence interval (SE = 0.019,95 % CI = [−0.089,−0.010]) so that H5a holds. Harmonious passion does not have a mediating effect between a diversified talent team construction and employee’s turnover intention. It contains 0 in the 95% confidence interval (SE = 0.009,95% CI = [−0.008,0.032]). So, H5b is invalid. Harmonious passion has a significant mediating effect between emphasis on employee’s training and employee’s turnover intention with a mediating effect of -0.036. It does not contain 0 in a 95% confidence interval (SE = 0.019, 95% CI = [−0.093,−0.008]). So, H5c holds. Emphasis on employee’s fairness and win–win can have a significant impact on the turnover intention of new generation employees through harmonious passion with an intermediary effect of −0.024. It does not contain 0 within a 95% confidence interval (SE = 0.009, 95% CI = [−0.065,−0.006]). So, H5d holds. In the relationship between emphasis on employee’s advantages and employee’s turnover intention, no significant mediating effect of harmonious passion was found. The 95% confidence interval (SE = 0.014, 95% CI = [−0.051,0.005]) contains 0, so H5e does not hold.

In addition, the test that obsessive passion is taken as a mediator found that there is no mediation of obsessive passion between the rational tolerance of employee’s opinions and failures and employee’s turnover intention. It contains 0 in the 95% confidence interval (SE = 0.014, 95% CI = [−0.005,0.051]). So, H6a is not valid. In the process of diversified talent team construction affecting the turnover intention of new generation employees, the obsessive passion did not play a significant mediating role. It contains 0 in a 95% confidence interval (SE = 0.012, 95% CI = [−0.012,0.038]). So, H6b is not true. Obsessive passion has a significant mediating effect in the process of emphasis on employee’s training affecting employee’s turnover intentions. It does not contain 0 in the 95% confidence interval (SE = 0.014, 95% CI = [−0.053, −0.009]), so H6c holds. Obsessive passion plays a mediating role between emphasis on fairness and win–win and the turnover intention of new generation employees. The mediating effect is −0.042 and it does not contain 0 within a 95% confidence interval (SE = 0.018, 95% CI = [−0.088,−0.016).]), so H6d is established. Obsessive passion plays a mediating role between emphasis on employee’s advantages and the turnover intention of new generation employees. The mediating effect is −0.067 and it does not contain 0 within a 95% confidence interval (SE = 0.026,95 % CI = [−0.130,−0.027]), so the H6e hold is valid.

## 5. Discussion

### 5.1. Contributions

#### 5.1.1. Theoretical Contribution

Firstly, our study enriches the relevant literatures of “human resource development model and employee’s turnover intention” of the past from the perspective of inclusive talent development model. Previous studies have focused on the influence of organizational support, organizational trust, organizational identity, psychological empowerment, organizational commitment and the exchanging relationship between leaders and employees on employee’s turnover intention [42,43,44,45,46,47]. These studies had not explored the influence of inclusive talent management model on the turnover intention of employees. Inclusive talent development model integrates the inclusive concept into the talent development model to improve employee’s ability, opportunity and motivation [17]. Our study has found the influence of the inclusive talent development model on employee’s turnover intention to be valid. It has important theoretical significance for the research of human resource development model and employee’s turnover intention behaviors.

Secondly, our study also enriches the theories of inclusive management and verifies the effectiveness of the inclusive talent development model through empirical research. In recent years, the academic community had carried out a lot of research on inclusive growth, inclusive development and inclusive leadership style [48,49,50,51]. However, the research of the inclusive talent development model is less. The inclusive talent development model is an important situational variable and offers an important perspective on diversity research in the theoretical framework of inclusive management. It has an important impact on employee’s behaviors. Therefore, our study, including research on the inclusive talent development model and empirical research on employee’s turnover intention, has great theoretical significance for improving and developing the theoretical system of inclusive management research. We found that the other four dimensions of the inclusive talent development model have an impact on employee’s work passion and turnover intention besides the diversified talent team dimension. The construction of a diversified talent team does not have a significant impact on employee’s work passion, nor does it have a significant inhibition on employee’s turnover intention. It is possible that a heterogeneous talent team will make members generate a certain conflict between ideas and behaviors [52]. This weakens employee’s perception of better working environment resources. That is why the construction of a diversified talent team does not have a significant impact on employee’s work passion and turnover intention.

Finally, “dualistic work passion” is introduced as an intermediary variable to verify the influence mechanism of the inclusive talent development model on employee’s turnover intention. This integrates and enriches the research theories of work passion, inclusive management and employee’s turnover. Previous studies on the influence of organizational behaviors and human resource management about employee’s turnover intention mainly focus on job satisfaction, employee’s well-being and job burnout as intermediary variables [42,44,53], which did not discuss the influence of work passion on employee’s turnover intention intensively. Our study examined the relationship between the inclusive talent development model, dualistic work passion and turnover intention of new generation employees. We found that the inclusive talent development model reduces turnover intention of new generation employees by improving the harmonious passion and reducing the obsessive passion. This finding extends the antecedent variables of dualistic work passion to the inclusive talent development model at the organizational level and shows the dualistic work passion’s effect on employee’s turnover intention.

#### 5.1.2. Practical Significance

Our study has an important guiding role in the practice of human resource management and provides reference and suggestions for the management of new generation employees. The conclusion of our study has the following implications for the practice of human resource management in enterprises.

Firstly, the concept of inclusion is introduced into the talent development model. It is an effective management model that affects the work passion of employees and reduces the turnover intention of new generation employees.

Secondly, enterprises should attach great importance to the issue of new generation employee’s passion and distinguishes two kinds of passion: harmonious passion has a negative impact on employee’s turnover intention, but obsessive passion has a positive impact on employee’s turnover intention.

Thirdly, enterprises should focus on the key dimensions of the inclusive talent development model that affect the work passion and turnover intention of new generation employees: rational tolerance of employee’s opinions and failures, emphasis on employee’s training, emphasis on fairness and win–win and emphasis on employee’s advantages. Management measures of these dimensions can significantly reduce turnover intention. Rational tolerance of employee’s opinions and failures, emphasis on employee’s training, emphasis on fairness and win–win can enhance the harmonious work passion. Emphasis on fairness and win-win and emphasis on employee’s advantages will reduce the obsessive passion of new generation employees.

#### 5.1.3. Research Limitations and Prospects

There are still some deficiencies in our study. First of all, the questionnaires collected in our study were filled in by employees. Although the statistical results had been verified to be free of serious common method bias, this problem cannot be completely excluded. In the future, multiple reports can be used to collect questionnaires to reduce the common method bias. Secondly, the research objects came from three provinces in East China. Although it has certain representativeness, the research scope still needs to be further expanded to make the research conclusion more generalizable. Thirdly, as a new type of inclusive management practice, the inclusive talent development model had not been paid attention to by the academic community. In the future, we will further explore the mechanism and boundary conditions of the influence of the inclusive talent development model on employee’s key work attitude, behaviors, ability and performance so as to enrich the theory of the inclusive talent development model.

### 5.2. Research Conclusion

Based on the self-determination theory and resource conservation theory, our study constructed a theoretical model of the mechanism of the inclusive talent development model on the work passion and turnover intention of new generation employees and found the following conclusions through empirical testing:

First of all, the inclusive talent development model has an important impact on the work passion and turnover behavior of new generation employees. Secondly, the two kinds of work passion have different effects on the turnover behaviors of new generation employees. Harmonious passion has a negative impact on the turnover intention of employees, but obsessive passion has a positive impact on the turnover intention of employees. Finally, work passion plays a mediating role between inclusive talent development and turnover behaviors of new generation employees. Harmonious passion plays a mediating role between rational tolerance of employee’s opinions and failures, emphasis on employee’s training and emphasis on fairness and win–win and employee’s turnover intention, while obsessive passion plays a significant mediating role between emphasis on employee’s training, emphasis on fairness and win–win and emphasis on employee’s advantages and employee’s turnover intention.

## 6. Conclusions

This study finds that inclusive talent development model requires diversified team building, tolerance of the viewpoints and shortcomings of staff, focus on employee engagement and fairness, and emphasis on employee advantages. The passion for work has been broken down into harmonious passion and obsessive passion. The work passion has been divided into harmonious passion and obsessive passion. Among them, rational tolerance of employee’s opinions and failures, emphasis on employee’s training and emphasis on fairness and win–win have a significant positive impact on harmonious passion. Emphasis on fairness and win–win and emphasis on employee’s advantages have negative correlation with obsessive passion. The harmonious passion of the employee is significantly correlated with the intention of turnover and the obsessive passion is significantly positively correlated with the intention of the turnover. Furthermore, the harmonious passion plays a mediating role between rational tolerance of employee’s opinions and failures, emphasis on employee’s training, emphasis on fairness and win–win and employee’s turnover intention, while the obsessive passion plays a mediating role between emphasis on fairness and win–win and emphasis on employee’s advantages and employee’s turnover intention.

## Figures and Tables

**Table 1:** Descriptive statistics of the study sample (*n* = 290).

Sociodemographic Characteristics		*n*	%
Gender	Male	156	53.8%
	Female	134	46.2%
Age	18–24	107	36.9%
	25–29	82	28.3%
	30–34	65	22.4%
	35–39	36	12.4%
Education level	Junior college or below	65	22.4%
	Bachelor’s	141	48.6%
	Master	60	20.7%
	Doctorate	24	8.3%
Working years	1 year and less	37	12.8%
	1–3 years	84	29.0%
	3–5 years	95	32.8%
	5–10 years	66	22.8%
	10 years and more	8	2.8%
Position	Top managers	9	3.1%
	Middle managers	30	10.3%
	Grassroots managers	105	36.2%
	Staff member	146	50.3%
Nature of enterprise	State-owned enterprise	78	26.9%
	Private enterprise	146	50.3%
	Foreign enterprise	38	13.1%
	Government-affiliated institutions	3	1.0%
	Other types	25	8.6%
Industry of the enterprise	Internet industry	65	22.4%
	Manufacturing industry	84	29.0%
	Software information service industry	35	12.1%
	Real estate	33	11.4%
	Finance and insurance	18	6.2%
	Consulting services	4	1.4%
	Scientific research and technical services	10	3.4%
	Other industries	41	14.1%

**Table 2:** The results of the reliability and convergence validity test.

Variables	Cronbach’s α	Factor Loading	CR	AVE
X1	0.885	0.830–0.864	0.885	0.720
X2	0.795	0.751–0.763	0.801	0.573
X3	0.817	0.739–0.814	0.819	0.601
X4	0.858	0.780–0.865	0.859	0.670
X5	0.855	0.700–0.919	0.861	0.677
HP	0.886	0.691–0.742	0.887	0.529
OP	0.883	0.697–0.727	0.884	0.522
Y	0.828	0.589–0.852	0.832	0.557
Suggested scope	>0.7	>0.6	>0.8	>0.5

Notes: (1) X1: rational tolerance of employee’s opinions and failures; X2: diversified talent team construction; X3: emphasis on employee’s training; X4: emphasis on fairness and win–win; X5: emphasis on employee’s advantages. HP: harmonious passion. OP: obsessive passion. Y: turnover intention. CR: Critical Ratio; AVE: Average Variance Extracted.

**Table 3:** The results of descriptive statistics and Pearson correlation coefficient.

Variables	Average	X1	X2	X3	X4	X5	HP	OP	Y
X1	3.37	0.849							
X2	3.24	0.410 ***	0.757						
X3	3.41	0.487 ***	0.359 ***	0.775					
X4	3.10	0.330 ***	0.329 ***	0.361***	0.819				
X5	2.95	0.426 ***	0.404 ***	0.413 ***	0.553 ***	0.823			
HP	3.07	0.516 ***	0.283 ***	0.508 ***	0.432 ***	0.432 ***	0.727		
OP	2.78	−0.177 ***	−0.181 ***	−0.272 ***	−0.469 ***	−0.517 ***	−0.341 ***	0.722	
Y	2.72	−0.395 ***	−0.222 ***	−0.487 ***	−0.411 ***	−0.438 ***	−0.466 ***	0.449 ***	0.746

Notes: (1) X1: rational tolerance of employee’s opinions and failures; X2: diversified talent team construction; X3: emphasis on employee’s training; X4: emphasis on fairness and win–win; X5: emphasis on employee’s advantages. HP: harmonious passion OP: obsessive passion. Y: turnover intention. (2) *** *p* < 0.001. (3) Diagonal bold font is AVE square root value; lower triangle is Pearson correlation.

**Table 4:** The results of regression analysis.

Dependent Variable→	HP		OP		Y			
Explanatory Variable↓	Equation	Equation	Equation	Equation	Equation	Equation	Equation	Equation
	1-1	1-2	2-1	2-2	3-1	3-2	4-1	4-2
Control variable								
Sexuality	−0.009	0.012	−0.120 *	−0.092	−0.029	0.005	−0.029	−0.037
Age	0.059	0.06	0.017	0.068	0.061	0.075	0.061	0.078
Education background	0.071	0.056	−0.008	−0.012	−0.024	0.001	−0.024	−0.018
Working years	−0.012	−0.01	−0.032	−0.025	−0.089	−0.083	−0.089	−0.091
Job grading	−0.018	−0.046	0.018	0.02	−0.001	−0.012	−0.001	0.017
Argument								
F1		0.270 ***		0.049				−0.119 *
F2		−0.017		0.109				0.003
F3		0.232 ***		−0.086				−0.253 ***
F4		0.171 **		−0.238 ***				−0.153 **
F5		0.098		−0.378 ***				−0.196 **
Mediation								
HP						−0.316 ***		
OP						0.308 ***		
Model Statistics								
R2	0.009	0.337	0.017	0.291	0.014	0.266	0.014	0.296
△R2	0.009	0.328	0.017	0.274	0.014	0.251	0.014	0.282
F-value	0.51	14.179 ***	0.97	11.464 ***	0.834	14.596 ***	0.834	11.749 ***

Notes: (1) F1: rational tolerance of employee’s opinions and failures; F2: diversified talent team construction; F3: emphasis on employee’s training; F4: emphasis on fairness and win–win; F5: emphasis on employee’s advantages. HP: harmonious passion. OP: obsessive passion. Y: turnover intention. (2) * *p* < 0.05, ** *p* < 0.01, *** *p* < 0.001.

**Table 5:** Mediation model fitting index table.

Index	CMIN/DF	RMSEA	CFI	TLI	SRMR
Value	1.397	0.037	0.960	0.956	0.050
Suggested scope	<3	<0.08	>0.9	>0.9	<0.05

Note: CMIN/DF: chi-square/ degree of freedom; RMSEA: Root Mean Square Error of Approximation; CFI: Comparative Fit. Index.; TLI: Tucker Lewis Index; SRMR: Standardized Root Mean Square Residual.

**Table 6:** The results of mediation test.

				95% Confidence Intervals	
Process	Estimate	S.E.	Est./S.E.	Upper Limit	Floor
F1→HP→Y	−0.038	0.019	−1.992	−0.089	−0.010
F2→HP→Y	0.005	0.009	0.545	−0.008	0.032
F3→HP→Y	−0.036	0.019	−1.843	−0.093	−0.008
F4→HP→Y	−0.024	0.014	−1.726	−0.065	−0.006
F5→HP→Y	−0.016	0.014	−1.151	−0.051	0.005
F1→OP→Y	0.015	0.014	1.089	−0.005	0.051
F2→OP→Y	0.009	0.012	0.714	−0.012	0.038
F3→OP→Y	−0.012	0.014	−0.800	−0.053	−0.009
F4→OP→Y	−0.042	0.018	−2.332	−0.088	−0.016
F5→OP→Y	−0.067	0.026	−2.544	−0.13	−0.027

Notes: (1) F1: rational tolerance of employee’s opinions and failures; F2: diversified talent team construction; F3: emphasis on employee’s training; F4: emphasis on fairness and win–win; F5: emphasis on employee’s advantages. HP: harmonious passion. OP: obsessive passion. Y: turnover intention. Est./S.E. = Estimate / S.E. (2) * *p* < 0.05, ** *p* < 0.01, *** *p* < 0.001.
